# 

**Published:** 1880-04

**Authors:** 


### Books and Pamphlets Received.

A System of Medicine. Edited by J. Russell Reynolds, M.D., F.R.S., with Numerous Additions and Illustrations by Henry Hartshorne, A.M., M.D. In three volumes. Vols. I and II received. Philadelphia: H. C. Lea. 1879.

The Causes and Results of Pulmonary Hæmorrhage with Remarks on Treatment. By Reginald E. Thompson, M.D. London: Smith, Elder & Co., 15 Waterloo Place. 1879.

The Hypodermic Injection of Morphia; Its History, Advantages and Dangers. By H. H. Kane, M.D. New York: Chas. L. Bermingham & Co. 1880.

Annual Report of the Supervising Surgeon-General of the Marine Hospital Service of the United States, for the Fiscal Years 1878 and 1879.

Proceedings of the Louisiana State Medical Association at its Second Meeting, Held in the City of New Orleans, April 9th, 10th and 11th, 1879, with the Constitution and by-laws.

Mansill's Almanac of Meteorology and Planetary Phenomena, for the Year 1880: and New System of Science. By Richard Mansill, Rock Island, Ill. R. Crampton, Publisher. 1880.

On the Internal Use of Water for the Sick, and on Thirst. A Clinical Lecture at the Pennsylvania Hospital, October 25, 1879. By J. Forsyth Meigs, M.D. Philadelphia: Lindsay and Blakiston. 1880.

Transactions of the Wisconsin State Medical Society, for the Year 1879, with its Constitution and By-Laws. Vol. XIII; 1879.

Transactions of the Medical Association of the State of Missouri, at its Twenty-second Annual Session, held in Columbia, Mo., May 20 and 21, 1879.

Transactions of the American Ophthalmological Society; Fifteenth Annual Meeting. Newport; 1879.

Primer of the Clinical Microscope. Boston Optical Works. By Ephraim Cutter, M.D.

Pacquelin's Thermo-Cautery with Wilson's Antithermic Shield in Epithelioma of the Cervix Uteri. By H. P. C. Wilson, M.D., Baltimore, Md.

Malignant Degeneration of a Fibroid Tumor of the Uterus. Large, False Aneurism in the Substance of the Growth. Drs. Albert N. Blodgett and Clifton E. Wing, Boston.

State Medicine and State Medical Societies. By Stanford E. Chaillé, A.M., M.D., New Orleans, Louisiana. Extracted from the transactions of the American Medical Association.

The Fallacies of Popular Clinical Medicine, an Introductory Lecture delivered at the Long Island College Hospital, Brooklyn, New York, February 5, 1880. By Jarvis S. Wright, M.D.

The New Anæsthetic—The Bromide of Ethyl. By R. J. Levis, M.D., Surgeon to the Pennsylvania Hospital, etc.

First Annual Report of the Central Board to the Philadelphia Society for Organizing Charitable Relief and Repressing Mendicancy. October 1, 1878.

The Second Annual Report of the Presbyterian Eye and Ear Charity Hospital, Baltimore, Md., for the year of 1879.

Report on the Revision of the U. S. Pharmacopœia, Preliminary to the Convention of 1880. By Charles Rice, Chairman of the Committee. New York: 1880.

---

NEW TREATMENT OF DIPHTHERIA.—M. A. Cornilleau. (*Rev. de Thér. Méd. Chir.* and *Rev. Méd.*, Jan. 24, 1880.)

The attention of the profession is called to the treatment of diphtheria with oxalic acid and oxalate of potassium.

The author's formula is as follows:

| | | Grams. | |
|---|---|---|---|
| Pure oxalic acid | (℈j) | 1 | 50 |
| Infusion of green tea | (℥iv) | 120 | |
| Syrup of bitter orange peel | (℥j) | 30 | |

to be taken in dessertspoonful doses every three hours.

In addition, to be taken every hour, a teacupful, more or less, according to the age, of the following:

| | | Grams. |
|---|---|---|
| Fresh sorrel leaves | (℥v) | 150 |
| Boiling water | (℥xxx) | 1000 |

To be sweetened at time of giving.

Speedy amelioration of the symptoms is observed under this treatment, convalescence being established generally at the end of a week.

The author has also found benefit from its use in croup. The dried leaves may be used when the fresh are not procurable.

### ANNOUNCEMENTS FOR THE MONTH.

### SOCIETY MEETINGS.

Chicago Medical Society—Mondays, April 5 and 19.

West Chicago Medical Society—Mondays, April 12 and 26.

### CLINICS.

MONDAY.

Eye and Ear Infirmary—2 p. m., Ophthalmological, by Prof. Holmes; 3 p. m., Otological, by Prof. Jones.

Mercy Hospital—2 p. m., Surgical, by Prof. Andrews.

Rush Medical College—2 p. m., Dermatological and Venereal, by Prof. Hyde; 3 p. m., Medical, by Dr. Bridge.

Woman's Medical College—2 p. m., Dermatological and Venereal, by Prof. Maynard; 3 p. m., Diseases of the Chest, Prof. Ingals.

TUESDAY.

Cook County Hospital—2 to 4 p. m., Medical and Surgical Clinics.

Mercy Hospital—2 p. m., Medical, by Prof. Hollister.

WEDNESDAY.

Chicago Medical College—2 p. m., Eye and Ear, by Prof. Jones.

Rush Medical College—3:30 to 4:30 p. m., Diseases of the Chest, by Dr. E. Fletcher Ingals.

THURSDAY.

Chicago Medical College—2 p. m., Gynæcological, by Prof. Jenks.

Rush Medical College—3 p. m., Diseases of the Nervous System, by Prof. Lyman.

Eye and Ear Infirmary—2 p. m., Ophthalmological, by Dr. Hotz.

Woman's Medical College—3 p. m., Surgical, by Prof. Owens.

FRIDAY.

Cook County Hospital—2 to 4 p. m., Medical and Surgical Clinics.

Mercy Hospital—2 p. m., Medical, by Prof. Davis.

SATURDAY.

Rush Medical College—2 p. m., Surgical, by Prof. Gunn.

Chicago Medical College—2 p. m., Surgical, by Prof. Isham; 3 p. m., Neurological, by Prof. Jewell.

Woman's Medical College—11 a. m., Ophthalmological, by Prof. Montgomery; 2 p. m., Gynæcological, by Prof. Fitch.

Daily Clinics, from 2 to 4 p. m., at the Central Free Dispensary, and at the South Side Dispensary.

